# Effects of dietary deoxynivalenol on growth performance and organ accumulation of growing pigs

**DOI:** 10.5713/ab.24.0177

**Published:** 2024-08-16

**Authors:** Ah Reum Son, Seung Youp Shin, Yoon Soo Song, Bokyung Hong, Beob Gyun Kim

**Affiliations:** 1 Department of Animal Science, Konkuk University, Seoul 05029, Korea

**Keywords:** Deoxynivalenol, Feeding Period, Growth Performance, Organ, Swine

## Abstract

**Objective:**

The present study aimed to study effects of a chronic feeding of deoxynivalenol (DON) on growth performance, organ weight, organ DON accumulation, and blood parameters in pigs.

**Methods:**

Forty-eight castrated male pigs with a body weight of 10.4 kg (standard deviation = 1.7) were assigned to one of 2 diet groups in a randomized complete block design with 6 blocks of pens per diet and 4 pigs per pen. A corn-soybean meal-based control diet was prepared to contain a low DON concentration of 0.28 mg/kg. Corn distillers dried grains with solubles naturally contaminated with DON were used at 30.0% to prepare a contaminated diet with a high DON concentration of 1.8 mg/kg. During the 56-day experimental period, body weight and feed intake were recorded every 14 days. A pig from each pen was euthanized for the collection of organs and muscle every 14 days.

**Results:**

Gain:feed in pigs fed the contaminated diet during days 14 to 28 and days 28 to 42 were less (p<0.05) compared with the control group. As increasing feeding period, the DON concentrations in fresh liver increased during days 14 to 28 and then decreased during the subsequent periods in the DON group, whereas the DON concentrations in fresh liver were constant during the experimental period in the control group (quadratic interaction p = 0.049). The DON concentration in the kidneys in the DON group was greater (p = 0.002) than that in the control group regardless of feeding period. On day 56, the granulocyte count in the DON group was less (p = 0.035) than the control group.

**Conclusion:**

A chronic feeding of DON for 14 to 42 days decreased gain:feed in pigs, and dietary DON naturally contaminated in corn distillers dried grains with solubles accumulated in the liver during days 14 to 28.

## INTRODUCTION

Deoxynivalenol (DON) is one of mycotoxins, which is generated by *Fusarium* fungus as secondary toxic metabolites in crops. The presence of DON in swine diets is mainly attributed to the cereal grains that are susceptible to DON contamination due to fungal growth before harvesting or during long-term storage in high-humidity conditions [1,2]. Cereal grain byproducts such as dried distillers grains with solubles (DDGS) can also be contaminated with DON due to the contamination of their substrate ingredients, of which concentrations may approximately 3 times compared with their substrate ingredients [3]. Pigs are highly sensitive to DON [4], resulting in decreased nutrient digestibility, impaired organs such as the liver and kidneys, and consequently, retarded growth performance in pigs [1,4–7]. Based on meta analyses, the voluntary feed intake of pigs is negatively affected by dietary DON [8,9].

Although DON is easily and rapidly absorbed and reaches peak of DON contents in plasma within 30 min following ingestion, DON is poorly metabolized or excreted [10,11], potentially resulting in a high retention rate of DON into tissues and organs of pigs. Goyarts et al [12] reported that pigs fed diets containing 6.68 mg DON/kg for 12 weeks showed more than 10 times of DON concentration in the kidneys and muscle compared with pigs fed a clean control diet. The accumulated DON in pig tissues and organs is potentially transferred into food chain and threats the human health. Moreover, DON accumulation in animal body tissues and organs can be affected by periods of feeding DON-contaminated diets even at low DON levels. However, very limited data on influence of feeding period of DON-contaminated diets to pigs are available. The objective of the present study, therefore, was to investigate the influences of dietary DON and feeding period on growth performance, organ weight, organ DON accumulation, and blood parameters in pigs. The hypothesis was that dietary DON would have adverse effects on the performance of pigs and would increase organ weight and accumulate in organs with feeding period.

## MATERIALS AND METHODS

### Animal care

All protocols of animal experiments were reviewed and approved by the Institutional Animal Care and Use Committee of Konkuk University (KU17120).

### Animals, diets, and experimental design

A total of 48 castrated male pigs (Duroc×[Landrace×Yorkshire]) with an initial body weight (BW) of 10.4 kg (standard deviation = 1.7) were randomly assigned to one of 2 diet groups in a randomized complete block design with BW as a blocking factor and with 6 blocks of pens per dietary treatment using a spreadsheet-based program [13]. Each pen consisted of 4 pigs. Two experimental diets were prepared: i) a control diet primarily consisted of corn, soybean meal, lactose, cellulose, and soybean oil and ii) a DON-contaminated diet formulated to contain 30.0% of corn DDGS naturally contaminated with DON to replace corn, soybean meal, cellulose, and soybean oil (Table 1). The DON-contaminated corn DDGS contained 5.2 mg/kg of DON, resulting in 1.8 mg/kg of DON in the contaminated diet (Table 2). Most limiting amino acid concentrations were formulated to be the same in both diets by supplementing crystalline amino acids to the diets. Vitamin and mineral concentrations in the experimental diets met or exceeded the requirement estimates suggested by the NRC [14]. A 2-hole feeder and a nipple drinker were installed in each pen with floor space of 2.0 m ×2.2 m. Pigs consumed water and feeds freely throughout the experiment.

### Feeding and sample collection

Individual BW and feed intake in each pen were recorded every 14 days to determine BW gain, feed intake, and gain-to-feed ratio (G:F). On days 14, 28, 42, and 56, a pig from each pen was euthanized to weight the organs of heart, liver, kidneys, and lungs and to collect the samples of the organs and longissimus dorsi muscle (LM). The collected samples were stored at −20°C. The organ weights were divided by BW to calculate organ weights relative to BW [15]. Blood samples were obtained on day 56 using ethylene diamine tetra-acetic acid tubes (Vacutainer No. 367844; Becton Dickinson, Franklin Lakes, NJ, USA) and were kept at 4°C before complete blood count analyses.

### Chemical analyses

Diets were finely ground to determine gross energy using an isoperibol bomb calorimeter (Parr 1261; Parr Instrument Co., Moline, IL, USA). Dry matter in diets was determined [16]. Crude protein (method 990.03), amylase-treated neutral detergent fiber inclusive of ash (method 2002.04), acid detergent fiber (method 973.18), and ash (method 942.05) in diets were analyzed according to the AOAC [17]. Deoxynivalenol concentrations in corn DDGS and the experimental diets were analyzed using enzyme-linked immunosorbent assay kits (AgraQuant, Romer Labs Inc., Singapore, Singapore) with quantification ranges for analysis on DON from 250 to 5,000 ng/mL. The complete blood count analysis was conducted immediately after collecting the blood samples using the HM2 (VetScan HM2 Hematology System; Abaxis, Union City, CA, USA). The samples of the liver, kidneys, and LM were dried using a forced-air laboratory drying oven at 105°C. The dried samples were finely ground to pass through a 1-mm screen and digested to determine DON concentrations according to the previously published procedure [18] with minor modification. Briefly, 0.2 g of weighed samples was transferred into a Pyrex screw cap glass tube and 2.5 mL of concentrated HNO_3_ and 0.5 mL of concentrated HCl was added. With the caps tightened, the tubes were placed in a water bath at 85°C for 3 h for digestion. After the digestion, the tubes were cooled down at room temperature, and the digested solution was then filtered using a 0.20 μm-pore diameter syringe filter. Double-distilled water was added to dilute each sample in a volumetric flask. After the digestion procedure, DON concentrations in the samples were determined using enzyme-linked immunosorbent assay kits (AgraQuant; Romer Labs Inc., Singapore, Singapore). The DON concentrations in fresh organs were calculated by multiplying the DON concentrations in dried samples with dry matter concentration in fresh organs.

### Statistical analyses

Experimental data were statistically analyzed using the MIXED procedure of SAS (SAS Inst. Inc., Cary, NC, USA). For growth performance, organ data, and complete blood count data, dietary treatment was regarded as a fixed effect and block as a random effect in the model: Y*_ij_* = *μ*+treatment*_i_*+ block*_j_*+ɛ*_ij_*; where Y is the response variable in the *i*th treatment and *j*th block, *μ* is the overall mean, and ɛ*_ij_* is the residual error. For the organ weight and DON concentration in organs or tissues, orthogonal contrasts were conducted to test the effects of dietary DON, feeding periods, and their interaction. The experimental unit was a pen for BW, average daily gain (ADG), average daily feed intake (ADFI), and G:F of pigs. An animal was regarded as an experimental unit for organ weight and DON concentration in organs or tissues as a pig from each pen was euthanized for the collection of organs and tissues on days 14, 28, 42, and 56. Statistical significance was declared at p<0.05 and statistical tendency was at p<0.10.

## RESULTS

During days 14 to 28 and days 28 to 42, the G:F of pigs fed the DON-contaminated diet was less (p<0.05) compared with that of pigs fed the control diet (Table 3). However, the differences in BW, ADG, and ADFI were not observed between the control group and the DON-contaminated group.

As increasing feeding period, the relative liver weight to BW was increased until day 28 and then decreased in pigs fed the DON-contaminated diet whereas the relative liver weight to BW was decreased in pigs fed the control diet (quadratic interaction p = 0.048; Table 4). The lung weights and relative lung weights to BW in pigs fed the DON-contaminated diet were greater (p<0.05) than those in pigs fed the control diet. With feeding period, lung weights linearly increased (p<0.05) but lung weights relative to BW linearly decreased (p<0.05) regardless of dietary DON.

As increasing feeding period, the DON concentrations in fresh liver were increased during days 14 to 28 and then decreased during the subsequent periods in the DON group, whereas the DON concentrations in fresh liver were constant during the experimental period in the control group (quadratic interaction p = 0.049; Table 5). Both concentration and weight of DON in the kidneys of pigs fed the DON-contaminated diet were greater (p<0.05) compared with those in pigs fed the control diet.

On day 56, the white blood cell count in pigs fed the control diet tended to be greater (p = 0.074; Table 6) than that in pigs fed the DON-contaminated diet. The lymphocyte count in pigs fed the control diet also tended to be greater (p = 0.096) than that in pigs fed the DON-contaminated diet. The granulocyte count in pigs fed the control diet was greater (p = 0.035) than that in pigs fed the DON-contaminated diet.

## DISCUSSION

Corn DDGS, a byproduct derived from the bioethanol production, are commonly used in swine diets due to the cost-effectiveness and the high protein concentration. Corn DDGS potentially contain a high concentration of DON likely due to corn contaminated with DON and undergoing concentration by approximately 3 times during fermentation processes [3]. In the present study, the DON-contaminated diet comprised 30.0% of corn DDGS containing 5.2 mg/kg of DON, resulting in a dietary DON concentration of 1.8 mg/kg which was close to the calculated value. The dietary DON concentration was relatively low compared with values in the previous studies [5,7,12,19,20] that investigated the effects of dietary DON on growth performance or various health parameters of pigs. However, long-term feeding with a low concentration of dietary DON can pose challenges to pig health and concomitant growth retardation [21,22]. More research is needed to better understand on the effects of feeding dietary DON on growth performance and health of pigs with increasing periods. In the present work, thus, the influence of dietary DON and feeding period on growth performance, organ weight, DON accumulation, and blood parameters was investigated in pigs.

The lack of responses in ADG and ADFI by dietary DON in the present work contrasts with previous studies [5,19,23, 24] that reported reduced feed intake and subsequent growth retardation in response to dietary DON. This inconsistency in the effects of dietary DON on growth performance is likely attributed to the relatively low DON concentration in diets of this study (1.8 mg DON/kg) compared with the high DON concentrations ranging from 3.0 to 6.4 mg/kg in the previous studies [5,19,23,24]. A tendency for reduced G:F by dietary DON during overall period observed in the present work agrees with the previous studies [8,9] and indicates that a low concentration of DON can negatively affect performance of pigs if DON is provided for an extended period. Ingested DON is rapidly absorbed into the plasma and distributed to various tissues and organs, potentially resulting in the accumulation of DON in the body and associated health impairments [12]. The accumulation of DON can limit protein synthesis by inhibiting the activity of peptidyl transferase on ribosomes in eukaryotic cells [4]. In the present work, no reduction in G:F by dietary DON was observed during the initial 14 days, suggesting that a diet with a low DON concentration may not immediately cause growth retardation in pigs. Instead, continuous ingestion of DON may lead to problems in subsequent periods. In a study by Prelusky et al [25], dietary DON at 3.0 mg/kg did not affect plasma alpha-globulin or cortisol concentrations in the pigs on day 18 of the experiment, but resulted in decreased plasma alpha-globulin concentrations and increased cortisol concentrations on day 32. These endocrine imbalances may be associated with clinical challenges in hematology and immune responses of animals as suggested by Prelusky et al [25].

While the relative liver weight to BW was decreased with feeding period in pigs fed the control diet, the relative liver weight to BW showed a quadratic response by feeding period with the greatest value on day 28 in pigs fed the DON-contaminated diet, indicating the quadratic interaction between dietary DON and feeding period effects. Ingested dietary mycotoxins are rapidly absorbed by pigs, but poorly metabolized or detoxified in the liver, which can increase the liver weight [8]. However, the reason for the quadratic response in the liver weight relative to BW in pigs fed the DON-contaminated diet remains unclear.

The relative weights of kidneys and heart to BW of pigs were not affected by DON consumption in the present work, which contrasts with the increased weights of kidneys and hearts by dietary DON in previous studies [8,12,26,27]. This inconsistency is also likely due to the relatively low concentration of dietary DON in this study, resulting in limited distribution of DON to the organs after absorption, unlike in the case of the liver. Conversely, increased relative lung weights to BW by consuming the low concentration of dietary DON were observed in the present work, but the reason for this remains unclear. The data for the effects of dietary DON on the relative lung weights to BW are very limited.

As the animals grew, the weight of heart, liver, kidneys, and lungs increased independently of dietary DON. However, the relative weight of organs to BW except for the kidneys decreased as the pigs grew regardless of dietary DON likely due to the relatively faster BW gain compared with organ weight increase, which is in agreement with the observations in a previous study [15].

While relatively constant DON concentrations were observed in the liver of pigs fed the control diet regardless of feeding period, a quadratic response for DON concentration in the fresh liver was observed in pigs fed the DON-contaminated diet with the greatest DON concentration on day 28. Interestingly, G:F and the liver weight relative to BW showed similar pattern in the present study. As discussed for the relative liver weight data, an increase in DON concentration in the liver can be due to the poor function of the liver for metabolizing and detoxifying toxins [8].

The greater DON concentration in the kidneys in the DON group compared with the control group is consistent with data in the literature [12,28]. Deoxynivalenol is primarily excreted through urine, leading to an increase in DON concentration in the kidneys after DON consumption [10,11]. Furthermore, Grenier et al [29] observed a greater incidence of kidney lesions associated with DON in pigs that consumed dietary DON compared with those in the control group. In the present study, however, the relative kidney weight to BW remained unaffected. This may be due to the accumulation of dietary DON in the kidneys during the urinary excretion process without reaching the toxicity level sufficient to enlarge the kidneys.

Although a quadratic response by feeding period was observed for the DON concentrations in the LM in the present study, the magnitude of changes is not that large. In addition, the DON concentrations in the liver, kidneys, and LM of pigs fed DON at 1.8 mg/kg for up to 56 days are less than the general DON allowance for human foods [30, 31]. However, it should be noted that a greater concentration of dietary DON may increase the DON in the organs and LM.

The decreases in the blood counts of white blood cell, lymphocyte, and granulocyte in this study are partially in agreement with Forsell et al [32] who reported the decreased leukocyte count along with reduced lymphocyte and monocyte counts in mice that consumed a DON-contaminated diet over 8 weeks. In other studies [20,26,29,33,34], the blood measurements were unaffected by feeding DON at 2.5 to 6.8 mg/kg to pigs for 28 to 84 days. Although the reason for this inconsistency remains unclear, different raising conditions for pigs and pig genotypes might have affected the response in the blood measurements.

## CONCLUSION

Feeding pigs deoxynivalenol-contaminated diets at 1.8 mg/kg led to a reduction in feed efficiency during days 14 to 42 and an increase in liver deoxynivalenol concentrations on day 28. The consumption of deoxynivalenol for 56 days resulted in decreased concentrations of white blood cells, lymphocytes, and granulocytes.

## Figures and Tables

**Table 1:** Ingredient composition of experimental diets (as-fed basis)^1)^

Ingredient (%)	Control diet	Contaminated diet
Ground corn	60.13	40.20
Soybean meal, 43% crude protein	27.00	22.00
Contaminated corn DDGS	-	30.00
Lactose	5.00	5.00
Cellulose	2.70	-
Soybean oil	2.00	-
Ground limestone	0.83	1.20
Dicalcium phosphate	1.14	0.60
L-Lys·HCl, 78.8%	0.39	0.40
DL-Met, 99.0%	0.12	-
L-Thr, 98.0%	0.09	-
Sodium chloride	0.30	0.30
Vitamin premix^2)^	0.10	0.10
Mineral premix^3)^	0.20	0.20

DDGS, distillers dried grains with solubles.

1) The contaminated corn DDGS contained 5.2 mg/kg of deoxynivalenol.

2) Provided the following concentrations of vitamins per kg of mixed diet: vitamin A, 12,500 IU; vitamin D_3_, 1,000 IU; vitamin E, 125 IU; vitamin K, 6.3 mg; thiamin, 6.3 mg; riboflavin, 25.0 mg; pyridoxine, 12.5 mg; vitamin B_12_, 0.1 mg; pantothenic acid, 100 mg; folic acid, 7.5 mg; niacin, 225 mg; biotin, 0.5 mg.

3) Provided the following concentrations of trace minerals per kg of mixed diet: Cu, 87.5 mg as copper sulfate; Fe, 125 mg as iron sulfate; I, 1.0 mg as potassium iodate; Mn, 75 mg as manganese sulfate; Se, 0.25 mg as sodium selenite; and Zn, 60 mg as zinc oxide.

**Table 2:** Calculated and analyzed composition of experimental diets (as-fed basis)

Items	Control diet	Contaminated diet
Calculated composition		
Metabolizable energy (kcal/kg)	3,305	3,302
Crude protein (%)	18.3	22.4
SID Lys (%)	1.13	1.13
SID Met (%)	0.37	0.33
SID Met+Cys (%)	0.62	0.62
SID Thr (%)	0.65	0.65
SID Trp (%)	0.19	0.19
SID Val (%)	0.71	0.86
Calcium (%)	0.68	0.68
STTD phosphorus (%)	0.32	0.32
Analyzed composition		
Dry matter (%)	88.7	88.8
Gross energy (kcal/kg)	3,972	3,979
Crude protein (%)	17.0	20.5
Amylase-treated neutral detergent fiber (%)	4.37	10.36
Acid detergent fiber (%)	0.61	1.01
Ash (%)	4.60	5.58
Deoxynivalenol (mg/kg)	0.28	1.80

SID, standardized ileal digestible; STTD, standardized total tract digestible.

**Table 3:** Effects of dietary deoxynivalenol on growth performance in pigs^1)^

Items	Control diet	Contaminated diet^2)^	SEM	p-value
Body weight (kg)				
Day 0	10.4	10.4	0.7	0.943
Day 14	16.1	16.4	1.2	0.467
Day 28	25.0	26.1	1.9	0.807
Day 42	39.2	38.7	2.5	0.613
Day 56	55.7	52.4	3.0	0.140
Days 0 to 14				
Average daily gain (g/d)	408	429	35	0.383
Average daily feed intake (g/d)	727	740	64	0.641
Gain:feed	0.559	0.576	0.010	0.231
Days 14 to 28				
Average daily gain (g/d)	675	653	57	0.412
Average daily feed intake (g/d)	1,226	1,243	97	0.665
Gain:feed	0.548	0.523	0.012	0.002
Days 28 to 42				
Average daily gain (g/d)	897	861	43	0.371
Average daily feed intake (g/d)	1,644	1,698	121	0.404
Gain:feed	0.550	0.512	0.017	<0.001
Days 42 to 56				
Average daily gain (g/d)	1,026	949	49	0.187
Average daily feed intake (g/d	2,189	2,248	146	0.688
Gain:feed	0.474	0.425	0.019	0.122
Days 0 to 56				
Average daily gain (g/d)	808	743	43	0.116
Average daily feed intake (g/d)	1,524	1,510	106	0.852
Gain:feed	0.534	0.494	0.013	0.062

SEM, standard error of the mean.

1) Data are least squares means of 6 replicate pens. Each pen consisted of 4 castrated male pigs per pen during day 0 to 14; an animal from each pen was euthanized on days 14, 28, and 42 resulting in less number of pigs per pen.

2) The contaminated diet contained 1.8 mg/kg of deoxynivalenol.

**Table 4:** Effects of dietary deoxynivalenol (DON) and feeding period on organ weight of pigs (wet basis)

Items	Control diet (d)				Contaminated diet^1)^ (d)				SEM	p-value^2)^				
		
	14	28	42	56	14	28	42	56		DON	L	Q	DON×L	DON×Q
No. of observations	5	6	6	6	6	6	6	6						
Organ weight (g)														
Heart	75	126	186	251	83	134	181	247	13	0.845	<0.001	0.362	0.478	0.951
Liver	423	631	941	1,346	391	696	1,033	1,272	63	0.666	<0.001	0.271	0.709	0.030
Kidneys	65	118	186	268	68	131	170	256	13	0.700	<0.001	0.098	0.305	0.861
Lungs	182	277	342	447	237	303	384	491	32	0.036	<0.001	0.507	0.908	0.690
Organ weight relative to body weight^3)^ (%)														
Heart	0.51	0.52	0.51	0.45	0.57	0.54	0.47	0.48	0.03	0.342	0.006	0.803	0.477	0.247
Liver	2.90	2.58	2.55	2.43	2.66	2.80	2.69	2.43	0.12	0.677	0.002	0.500	0.336	0.048
Kidneys	0.46	0.48	0.50	0.48	0.47	0.52	0.45	0.49	0.03	0.891	0.527	0.433	0.580	0.549
Lungs	1.27	1.17	0.93	0.82	1.64	1.21	1.01	0.94	0.10	0.010	<0.001	0.130	0.178	0.101

SEM, standard error of the mean.

1) The contaminated diet contained 1.8 mg/kg of DON.

2) DON, dietary DON contamination; L, linear effect of feeding period; Q, quadratic effect of feeding period; DON×L, interaction between dietary DON contamination and linear effect of feeding period; DON×Q, interaction between dietary DON contamination and quadratic effect of feeding period.

3) Relative organ weights to body weight (%) = (organ weight [kg] / body weight [kg])×100.

**Table 5:** Effects of dietary deoxynivalenol (DON) and feeding period on DON concentration of pig organs (wet basis)

Items	Control diet (d)				Contaminated diet^1)^ (d)				SEM	p-value^2)^				
		
	14	28	42	56	14	28	42	56		DON	L	Q	DON×L	DON×Q
No. of observations	5	6	6	6	6	6	6	6						
DON concentration in fresh tissue^3)^ (mg/kg)														
Liver	0.062	0.082	0.059	0.081	0.070	0.132	0.073	0.074	0.012	0.040	0.864	0.059	0.240	0.049
Kidneys	0.076	0.068	0.047	0.050	0.111	0.081	0.117	0.064	0.014	0.002	0.026	0.752	0.933	0.391
LM	0.038	0.031	0.019	0.037	0.038	0.038	0.032	0.038	0.005	0.157	0.464	0.037	0.775	0.205
DON weight in fresh organ (mg)														
Liver	0.027	0.050	0.053	0.105	0.027	0.092	0.073	0.094	0.011	0.118	<0.001	0.638	0.462	0.030
Kidneys	0.005	0.008	0.009	0.012	0.008	0.012	0.021	0.016	0.003	0.002	0.001	0.235	0.410	0.212

SEM, standard error of the mean; LM, longissimus dorsi muscle.

1) The contaminated diet contained 1.8 mg/kg of DON.

2) DON, dietary DON contamination; L, linear effect of feeding period; Q, quadratic effect of feeding period; DON×L, interaction between dietary DON contamination and linear effect of feeding period; DON×Q, interaction between dietary DON contamination and quadratic effect of feeding period.

3) The DON concentrations in fresh organs were calculated by multiplying the DON concentrations in dried samples with dry matter concentration in fresh organ.

**Table 6:** Effects of dietary deoxynivalenol on complete blood count traits on day 56 in pigs^1)^

Items	Control diet	Contaminated diet^2)^	SEM	p-value
White blood cell (10^9^/L)	21.0	18.3	2.1	0.074
Red blood cell (RBC; 10^9^/L)	7.32	7.59	0.29	0.532
Hemoglobin (g/dL)	11.7	12.3	0.6	0.491
Hematocrit (%)	36.0	38.0	1.5	0.375
Mean corpuscular volume (fL)	49.3	50.0	1.0	0.613
RBC distribution width (%)	20.4	20.4	0.4	0.941
Mean corpuscular hemoglobin (MCH; pg)	16.0	16.2	0.3	0.674
MCH concentration (g/dL)	32.6	32.4	0.4	0.600
Platelet (PLT; 10^9^/L)	272	310	35	0.457
Mean PLT volume (fL)	9.68	9.61	0.23	0.803
PLT percentage (%)	0.26	0.30	0.03	0.376
PLT distribution width concentration (%)	39.2	39.1	0.5	0.834
Lymphocyte (10^9^/L)	20.5	17.7	2.1	0.096
Monocyte (10^9^/L)	0.33	0.38	0.15	0.803
Granulocyte (10^9^/L)	0.22	0.18	0.02	0.035

SEM, standard error of the mean.

1) A pig in each pen was bled and data are least squares means of 6 observations for all treatment.

2) The contaminated diet contained 1.8 mg/kg of deoxynivalenol.
